# Vitamin D and Reproduction: From Gametes to Childhood

**DOI:** 10.3390/healthcare3041097

**Published:** 2015-11-09

**Authors:** Krista D. Sowell, Carl L. Keen, Janet Y. Uriu-Adams

**Affiliations:** 1 Department of Nutrition, University of California, Davis, One Shields Avenue, Davis, CA 95616, USA; E-Mails: kdsowell@ucdavis.edu (K.D.S.); clkeen@ucdavis.edu (C.L.K.); 2 Department of Internal Medicine, University of California, Davis One Shields Avenue, Davis, CA 95616, USA

**Keywords:** vitamin D, vitamin D supplementation, pregnancy, fertility, toxicity, preeclampsia, gestational diabetes, allergic disease

## Abstract

Vitamin D is well recognized for its essentiality in maintaining skeletal health. Recent research has suggested that vitamin D may exert a broad range of roles throughout the human life cycle starting from reproduction to adult chronic disease risk. Rates of vitamin D deficiency during pregnancy remain high worldwide. Vitamin D deficiency has been associated with an increased risk of fertility problems, preeclampsia, gestational diabetes, and allergic disease in the offspring. Vitamin D is found naturally in only a few foods thus supplementation can provide an accessible and effective way to raise vitamin D status when dietary intakes and sunlight exposure are low. However, the possibility of overconsumption and possible adverse effects is under debate. The effect of vitamin D supplementation during pregnancy and early life on maternal and infant outcomes will be of particular focus in this review.

## 1. Introduction

While the beneficial effects of vitamin D supplementation with respect to reducing the risk of rickets has been recognized for close to 100 years, there is increasing concern that rickets in young children is reemerging in numerous countries [1,2]. In the U.S. today, a disproportionate percentage of children with rickets are African American, who are characterized by low rates of vitamin D synthesis, and exclusively breast-fed infants, who are characterized by low vitamin D intakes. Over the past two decades, it has been well accepted that vitamin D is a pro-hormone, and suboptimal vitamin D status has been increasingly linked to a broad range of diseases including, cancer, diabetes, cardiovascular disease, and autoimmune disorders. Given the above, during the past decade there has been a marked increase in research on the potential health effects of this “vitamin” [3].

Currently, it is recognized that vitamin D deficiency can be demonstrated in numerous ethnic populations and locations around the world. However, the prevalence of vitamin D deficiency is a subject of intense debate, in part due to differences of opinion regarding what actually constitutes a “deficiency”. Illustrative of the above, are the markedly different circulating vitamin D concentration cut-offs for the risk of vitamin D deficiency, insufficiency, and sufficiency levels that have been identified by the U.S. Institute of Medicine (IOM) and the U.S. Endocrine Society (Table 1). While the incidence of vitamin D deficiency in most countries would seem to be moderate if the IOM cut-offs are used, the incidence is high in many countries if the cut-off values advanced by the Endocrine Society are embraced [3]. Vitamin D deficiency is common among women in the United States with 35% of women between 20 and 49 years of age having vitamin D concentrations below 50 nmol/L (the IOM cut-off for vitamin D sufficiency) [4]. This number increases to 82% in African Americans and 58% in Mexican Americans. Estimates of vitamin D deficiency during pregnancy worldwide range from 8% to 99% of women depending on the population and cut-offs used. Rickets will not be discussed in this review (readers are referred to a review on this topic by Elder and Bishop, 2014 [5]) as this paper is focused on recent literature regarding the importance of maternal vitamin D status on pregnancy and infant outcomes, with an emphasis on supplementation trials.

**Table 1:** Designation of vitamin D status based on circulating vitamin D levels by the U.S. Institute of Medicine (IOM) and the Endocrine Society.

Vitamin D Status	IOM	Endocrine Society
Deficiency	<12 ng/mL (30 nmol/L)	<20 ng/mL (50 nmol/L)
Insufficiency	12–20 ng/mL (30–50 nmol/L)	21–29 ng/mL (52.5–72.5 nmol/L)
Sufficiency	>20 ng/mL (50 nmol/L)	>30 ng/mL (75 nmol/L)
Toxicity	>50 ng/mL (125 nmol/L)	>150 ng/mL (375 nmol/L)

## 2. Factors Influencing Vitamin D Status

**Figure 1:**
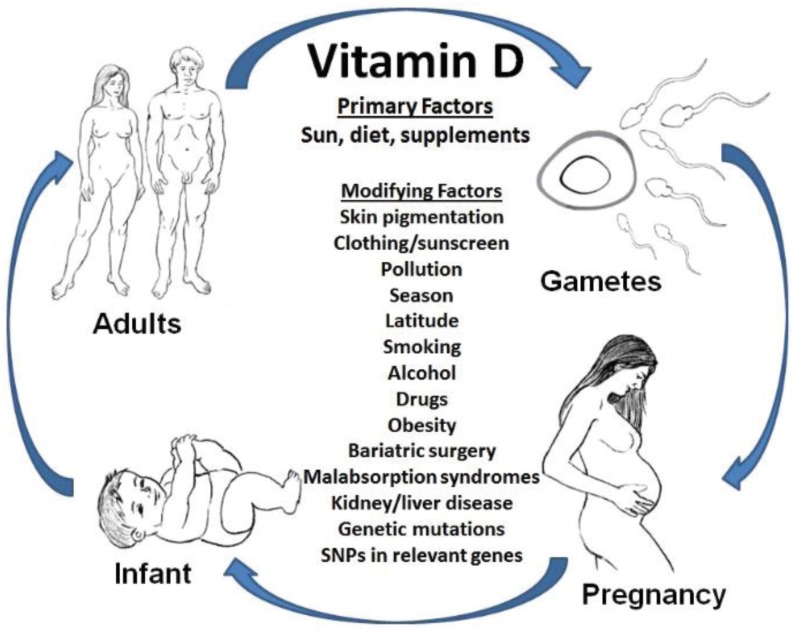
Modifying factors that can affect vitamin D status throughout the life cycle.

Numerous and diverse lifestyle choices, and environmental and genetic factors can alter vitamin D status [6]; Figure 1. Vitamin D in the human body can arise in two different ways: (1) secondary to ultraviolet B (UVB) radiation exposure and (2) the dietary consumption of the vitamin. UVB radiation penetrates the skin epidermis to convert 7-dehydrochesterol into previtamin D_3_. Differences in the degree of skin melanin can modify UVB-induced production of vitamin D by over 90%, putting dark skinned individuals at higher risk for a deficiency. Increased time spent indoors, dermatologist recommendations of daily sunscreen application, geographical location (northern latitude) and seasonal changes (winter) can reduce exposure to UVB radiation and subsequently lower vitamin D synthesis leading to lower vitamin D levels [6].

Previtamin D_3_ isomerizes into vitamin D_3_ (cholecalciferol) and is subsequently metabolized by 25-hydroxylase (CYP2R1) to 25-hydroxyvitamin D_3_ (25(OH)D) in the liver. Vitamin D binding protein transports the 25(OH)D to the kidneys where hydroxylation by 1-α-hydroxylase to the active form, 1,25-dihydroxyvitamin D_3_ (1,25(OH)_2_D) occurs. Genetic variations in CYP2R1 and vitamin D binding protein can reduce the subsequent increases of serum vitamin D after UVB exposure and consumption of vitamin D fortified foods, respectively, suggesting genetics play a role in determining vitamin D status [7]. High levels of body fat can sequester vitamin D into adipose cells thus reducing its bioavailability to other tissues. Given the above it has been argued that obese individuals are a population that is at an increased risk for vitamin D deficiency [3,8]. Consistent with this, it has been reported that obese pregnant women have lower vitamin D levels than non-obese women, even when they have higher vitamin D intakes during the first trimester [9]. Based on lower circulating 25(OH)D concentrations at a given vitamin D supplement level in obese and overweight subjects compared to normal weight subjects, Ekwaru and coworkers recently recommended that “vitamin D supplementation be 2–3 times higher for obese subjects and 1.5 times higher for overweight subjects relative to normal weight subjects” [10].

There are two forms of vitamin D that are important in humans. Vitamin D_2_ (ergocalciferol), is made by plants and vitamin D_3_, is made by sunlight exposure on skin. It has been thought that vitamins D_2_ and D_3_ are equivalent as increases in circulating 25(OH)D levels were found to be similar in subjects after an 11-week supplementation of 1000 IU/day of vitamin D_2_ or D_3_ [11]. In contrast, differences in 25(OH)D concentrations are seen following a one-time oral 50,000 IU bolus of D_2_ or D_3_ [12]. The increase in serum 25(OH)D was identical between the two groups in the first three days following supplementation, however, the D_2_-treated subjects had a decrease in 25(OH)D levels reaching baseline levels by day 14, while levels continued to increase in D_3_-treated subjects at day 14 and remained higher at day 28 than D_2_-treated subjects. These studies show that the pharmacokinetics of low doses of D_2_ and D_3_ may be equivalent, but at larger doses, vitamin D_2_ might be less effective than vitamin D_3_ at sustaining 25(OH)D levels.

Today, vitamin D is thought to have multiple functions beyond its role(s) in bone health [13]. Illustrative of this, vitamin D nuclear receptors (VDR) have been identified in numerous tissues including organs involved in reproduction and infant growth such as the ovary, testis, placenta and mammary gland [14,15]. Once bound to vitamin D, VDR can form a heterodimer with retinoid-x receptor and bind to response elements on promoter regions of DNA leading to changes in the transcription of specific target genes. Multiple tissues express 1-α-hydroxylase which can produce 1,25(OH)_2_D locally, provided there is an adequate 25(OH)D supply available [14].

## 3. Vitamin D and Reproduction

Vitamin D is important for all stages of the life cycle. Circulating levels of vitamin D are used as biomarkers of exposure combining both production from UVB exposure and dietary intake rather than as biomarkers of effect. The extent to which circulating 25(OH)D levels directly relate to specific health outcomes is an active area of research. The current review is focused on the effects of vitamin D on numerous aspects of reproduction including male and female fertility, pregnancy, and infant outcomes as well as childhood and adult disease (Figure 1).

### 3.1. Male Fertility

Infertility is a devastating situation that many couples face. Convincing data from animal models show that vitamin D deficiency or altered function can result in reduced liter size or loss of overall fertility in conjunction with an increase in maternal and offspring mortality [16,17]. The reductions in fertility can be restored by vitamin D supplementation. Vitamin D has been implicated in sperm motility and development in animals and humans [18]. VDR and enzymes necessary for vitamin D metabolism have been found in male testis, mature spermatozoa, and the ejaculatory tract suggesting an important role of this vitamin in male fertility [19]. Male rats fed a vitamin D deficient diet prior to mating were 17% less likely to inseminate females than vitamin D replete rats [20]. Only 40% of the females inseminated by vitamin D deficient rats had normal sized litters compared to 76% from vitamin D-replete males in part due to increased pregnancy complications. VDR null male mice exhibit decreased estrogen levels, sperm count, and motility compared to wild-type mice [21]. Supplementation with estrogen was able to prevent the histological changes to VDR-null male testis and normalize follicular stimulating hormone (FSH), and luteinizing hormone (LH) levels, indicating vitamin D might have a contributing role in spermatogenesis and male fertility. In agreement, epidemiological studies have shown that men with deficient vitamin D levels (using either the IOM cut-off of <25 nmol/L [22] or the Endocrine Society cut-off of <50 nmol/L [23]) have reduced sperm motility, progressive motility, and number of mature spermatozoa.

### 3.2. Female Fertility

VDR knockout female mice have altered synthesis of estrogen, FSH, and LH [16], all hormones essential for reproductive success. Johnson *et al.*, restored fertility and litter size by feeding a high calcium diet to female VDR null mice suggesting a plausible role of vitamin D in calcium homeostasis rather than a direct effect on VDR [17]. Vitamin D is further linked to embryo implantation in the uterus. HOXA10, a gene important in human stromal cells for embryo implantation, is upregulated by VDR and estrogen levels signifying a possible link to favorable pregnancy outcomes [24].

Ota *et al.*, reported that 47.4% of women with recurrent pregnancy losses (RPL), defined as three or more consecutive spontaneous abortions before 20 weeks gestation, had low serum 25(OH)D levels e.g., below 30 ng/mL, the Endocrine Society cut-off for vitamin D sufficiency status (Table 1) [25]. Compared to women with serum levels ≥30 ng/mL, women with low vitamin D status had higher circulating antiphospholipid antibodies associated with poor reproductive outcomes. Women with RPL have higher natural killer (NK) cell cytotoxicity compared to control women, and in an *in vitro* model, 1,25(OH)_2_D was able to modify NK cell function and reduce their cytotoxicity [26]. In a recent study, Anderson *et al.*, reported that Danish women (Odense Child Cohort) with 25(OH)D levels < 50 nmol/L (IOM cut-off for vitamin D sufficiency and Endocrine Society cut-off for deficiency, Table 1) had a greater than 2-fold increased adjusted hazards ratio for first-trimester miscarriage [27]. In contrast, Mollor *et al.*, detected no difference in pre-pregnancy vitamin D status among healthy Danish women who were able to conceive compared to those who could not conceive in a ~6 month time period [28]. However, women who had a miscarriage after the 10th week of gestation had lower plasma 25(OH)D levels in their first trimester compared to women who had live births (36 nmol/L and 65 nmol/L, respectively, *p* = 0.03).

The use of *in vitro* fertilization (IVF) has enabled insight into fertility research. Many observational studies have linked higher maternal vitamin D status to an increase in rates of both clinical pregnancy (sonographic presence of a heartbeat following IVF) and live birth [29,30,31]. Ethnic differences were observed by Rudick *et al.*, with a positive relationship of maternal 25(OH)D and post-IVF pregnancy rates for non-Hispanic whites, but the opposite was found in Asian women [31,32]. Other reproductive outcomes, including ovarian stimulation, fertilization rates, or markers of embryo quality were not affected by varying vitamin D concentrations or race. This indicates vitamin D can have a positive impact on the endometrium and subsequent embryo implantation, an idea supported by animal models [32]. The positive relationship between maternal vitamin D status and post-IVF pregnancy in non-Hispanic white, but not in Asian women, suggests that genetics play a role in the relationship between vitamin D and fertility. This finding could impact doctor’s recommendation of what are considered optimal vitamin D levels when women of different ethnic groups undergo IVF.

A supplementation trial involving 128 vitamin D insufficient (<30 ng/mL, as classified by the authors) Iranian women six to eight weeks prior to undergoing freeze-thaw embryo transfer found no differences in pregnancy rates between women receiving 50,000 IU vitamin D/week compared to women receiving placebo [33]. All women receiving the weekly supplement reached vitamin D sufficiency as assessed by both IOM and Endocrine Society cut-offs (25(OH)D) = 47.65 ± 1.11 ng/mL) at the end of the study. The population in the study represented a homogenous population of women with vitamin D insufficiency at baseline (15.81 ± 5.94 ng/mL). Additional clinical trials in ethnically diverse populations with differing baseline vitamin D levels are needed to better delineate the effect of vitamin D status on assisted reproductive technology outcomes.

In an ongoing clinical trial (PREPARE trial), the effects of a “Mediterranean” diet are being tested on multiple markers of early embryo development, implantation and pregnancy rates [34]. The supplemental diet which includes olive oil for cooking, an olive oil based spread, and a daily supplement drink enriched with Vitamin D (400 IU) and the marine omega-3 fatty acids EPA (1 g) and DHA (1 g) is provided to male and female partners for six weeks prior to IVF-induced conception. The control diet includes sunflower seed oil for cooking, a sunflower oil based spread, and a daily supplement drink without vitamin D, EPA or DHA. Although the amount of vitamin D provided through the supplement is lower than the current RDAs set by either the IOM or Endocrine Society, this study should provide new insights into how periconceptional dietary interventions can impact female and male fertility, a research area that is presently lacking.

## 4. Pregnancy

The 2004–2008 National Health and Nutrition Examination Survey (NHANES) data shows that the cumulative frequency of vitamin D deficiency during pregnancy in all races is 29% according to Endocrine Society criteria (< 20 ng/mL). When separated into ethnic groups, frequencies of vitamin D deficiency are highest in African Americans (75%), followed by Mexican-Americans (42%) and non-Hispanic whites (9%) [4]. Evaluation of NHANES data from 2001 to 2006 using IOM guidelines reports that 7% of pregnant women are vitamin D deficient (< 12 ng/mL) and 21% are vitamin D insufficient (12–20 ng/mL) [35]. These data further illustrate how different cut-offs used by the IOM *vs.* Endocrine Society for defining vitamin D status (Table 1) influences the reporting of vitamin D deficiency prevalence in the population. The current RDA for vitamin D is 600 IU/day for both non-pregnant females of reproductive age and pregnant/lactating females according to IOM recommendations (Table 2). The IOM RDA is set to meet the total intake requirement of 97.5% of healthy individuals with minimal or no sunlight exposure. In contrast to IOM recommendations, the Endocrine Society recommends a vitamin D intake of at least 600 IU/day, but states that 1500–2000 IU/day may be needed for pregnant or lactating females to maintain a 25(OH)D level ≥ 30 ng/mL.

While 25(OH)D remains constant, the concentrations of total 1,25(OH)_2_D and vitamin D binding protein increase in the first to second trimester and remain elevated until 3 months postpartum [36]. Vitamin D functions to modulate the immune system by heightening the innate system and muting the adaptive immune system. During pregnancy, this should aid in increasing maternal ability to fight off infections and creating fetal-tolerance to avoid rejection, respectively [37]. Under normal conditions, activation of toll like receptors (TLR) on the immune cell’s surface triggers the expression of VDR and CYP27B1, increasing local concentrations of 1,25(OH)_2_D_3_ [37,38,39]. Dendritic cells are the main immune cells responsible for connecting the innate and adaptive immune systems and are vital for T cell proliferation. Vitamin D can inhibit dendritic cell differentiation and maturation increasing the formation of tolerogenic dendritic cells [40]. The altered cytokine production in these cells leads to a shift in the differentiation of naïve T cells from T_H_1 to T_H_2, wherein a weighted T_H_2 response is associated with fetal tolerance, and regulatory T cells. Regulatory T cells are important in modulating the immune system to become more tolerate to self-antigens and function to suppress further immune activation. Cathelicidins, antimicrobial peptides that can defend against pathogens, are up regulated by vitamin D. VDR can bind to response elements of the human cathelicidin peptide gene (cathelicidin antimicrobial peptide; CAMP) increasing the killing ability of innate immune cells, macrophages, thus strengthening the innate immune system’s response [41]. These changes induced by vitamin D would dampen the adaptive immune system while optimizing the innate immune’s function to kill pathogens aiding the maternal body to protect against microbial insults and establish an environment of fetal tolerance.

**Table 2:** Vitamin D recommended intakes.

Age	IOM		Endocrine Society		Australian NHMRC **		WHO
	RDA	UL	Daily Requirement	UL	AI	UL	RNI
0–6 months	400 IU (10 μg) *	1000 IU (25 μg)	400–1000 IU	2000 IU (50 μg)	200 IU (5 μg)	1000 IU (25 μg)	200 IU (5 μg)
7–12 months	400 IU (10 μg) *	1500 IU (38 μg)	400–1000 IU	2000 IU (50 μg)	200 IU (5 μg)	1000 IU (25 μg)	200 IU (5 μg)
1–3 years	600 IU (15 μg)	2500 IU (63μg)	600–1000 IU	4000 IU (100 μg)	200 IU (5 μg)	3200 IU (80 μg)	200 IU (5 μg)
4–8 years	600 IU (15 μg)	3000 IU (75 μg)	600–1000 IU	4000 IU (100 μg)	200 IU (5 μg)	3200 IU (80 μg)	200 IU (5 μg)
Pregnancy/Lactation							
14–18 years	600 IU (15 μg)	4000 IU (100 μg)	600–1000 IU	4000 IU (100 μg)	200–400 IU	3200 IU (80 μg)	200 IU (5 μg)
19–50 years	600 IU (15 μg)	4000 IU (100 μg)	1500–2000 IU	10,000 IU (250 μg)	200–400 IU	3200 IU (80 μg)	200 IU (5 μg)

* Adequate Intake (AI); ** National Health and Medical Research Council.

### 4.1. Gestational Diabetes

The impact of Vitamin D on vascular health and glucose control can likewise translate to adverse effects of vitamin D deficiency on pregnancy outcomes including an increased risk for gestational diabetes and preeclampsia. Low vitamin D has been associated with insulin resistance. The theory that vitamin D supplementation can help normalize blood glucose levels and reduce the incidence of gestational diabetes is an active area of research. First trimester 25(OH)D levels were found to be lower in women diagnosed with gestational diabetes compared to controls, translating to a 2.6-fold increased risk of developing gestational diabetes when serum vitamin D levels were ≤20 ng/mL, the IOM cut-off for vitamin D sufficiency [42]. After restricting the analysis to non-Hispanic whites (the majority of the study population), a 3.7-fold increased risk of gestational diabetes was seen. However, this association with an increased risk of gestation diabetes with low 25(OH)D levels is not consistent across observational studies [43,44].

Gestational diabetes is associated with insulin resistance, an increase in inflammation and subsequent rise in oxidative stress. The effects of vitamin D supplementation (400 IU /day vitamin D_3_) on insulin and glucose metabolism were observed in healthy, pregnant women in their 25th week of gestation [45]. After nine weeks of supplementation of vitamin D or placebo, the vitamin D supplemented group had reductions in serum insulin, fasting plasma glucose levels, high sensitivity C-reactive protein (hs-CRP), and blood pressure compared to the placebo group. Although positive effects were seen, 41.7% of the participants in the intervention group and 83% in the placebo group remained vitamin D deficient (using the Endocrine Society cutoff; 20.8% and 54.2%, respectively using IOM guidelines) due to the low intervention dose.

In a 2013 randomized controlled trial, Asemi *et al.*, provided 50,000 IU vitamin D_3_ or placebo at baseline and day 21 of the six week trial to women diagnosed with gestational diabetes in their 24th–28th week of gestation [46]. After six weeks, there was a significant increase in serum 25(OH)D in the vitamin D group (+18.51 ± 20.46 ng/mL). Improved serum insulin, insulin resistance index (HOMA-IR), fasting plasma glucose, LDL and total cholesterol were noted in the vitamin D group compared to the placebo group. With regard to pregnancy outcome, there were no cases of polyhydramnios (the accumulation of excess amniotic fluid) in the vitamin D group compared to 17.4% in the placebo group, and a marked reduction of hyperbilirubinemia in infants from the vitamin D supplemented group (27.3%) compared to the placebo group (60.9%) was reported (*p* = 0.02) [47]. These data suggest a positive effect of vitamin D supplementation on pregnancy outcomes in women with gestational diabetes.

Asemi *et al.*, investigated the effects of vitamin D co-supplemented with calcium on metabolic parameters in women diagnosed with gestational diabetes starting in their 24th–28th week of gestation. Women were provided 1000 mg/day calcium and a bolus of 50,000 IU of vitamin D_3_ twice during the six week study, one at baseline and one at intervention day 21, or placebo [48]. After the six weeks, a significant increase in serum 25(OH)D (+48.91 ± 46.64 ng/mL) was observed in the treatment group. In agreement with another study by the same group [45], vitamin D plus calcium supplementation improved fasting plasma glucose, serum insulin, HOMA-IR, HDL, and LDL in women with gestational diabetes compared to placebo. Vitamin D supplementation also resulted in an increase in plasma glutathione (antioxidant) and mitigated the rise in malondialdehyde (a biomarker for lipid oxidative damage), although no change was noted in hs-CRP levels. Given the heightened response with co-supplementation, it suggests that the effects of vitamin D on gestational diabetes might be enhanced with the use of calcium. While vitamin D supplementation to women with gestational diabetes improved metabolic parameters, what is needed are clinical trials investigating the extent to which vitamin D supplementation decreases adverse perinatal outcomes associated with gestational diabetes including macrosomia, respiratory distress, and postnatal hypoglycemia.

To test the dose response effect of vitamin D on glucose metabolism, doses of 200 IU/day, 50,000 IU/monthly, or 50,000 IU/biweekly vitamin D_3_ were chosen to supplement healthy (non-gestational diabetes), pregnant women with severe vitamin D deficiency (7.6 ± 6.3 ng/mL) from the 12th week of gestation to delivery [49]. According to Endocrine Society cut-offs (Table 1), the biweekly high dose group was able to achieve sufficiency in 62.5% of subjects, the high dose monthly group had 39.5% of subjects while 11.4% of subjects in the low dose (200 IU/day) had sufficient vitamin D levels. There was a dose response decrease in the rise of serum insulin and HOMA-IR with increasing vitamin D supplementation. Serum calcium was similar among the groups further suggesting no adverse side effects with the vitamin D dosages tested.

### 4.2. Preeclampsia

Risk factors for preeclampsia include age, obesity, first time pregnancy, and prior history of preeclampsia. The occurrence of gestational diabetes is associated with an increased risk for severe or mild preeclampsia and gestational hypertension. Low maternal 25(OH)D levels have been associated with a greater risk of preeclampsia in a number of studies [50,51,52,53]. A 27% reduction in the risk of preeclampsia was found in women who self-reported taking 400–600 IU vitamin D_3_ supplements per day compared to women who did not [54]. It should be noted that other studies have failed to confirm observations that lower 25(OH)D status is associated with the occurrence of preeclampsia, and it is still unclear whether suboptimal vitamin D status is a cause, or an effect, of preeclampsia [55,56].

Anti-inflammatory and immune modulating properties of vitamin D are postulated to contribute to the protective mechanism against preeclampsia. A recent study by Darby *et al.*, investigated the association of circulating maternal 1,25(OH)_2_D levels on preeclampsia and the effect of vitamin D supplementation on placental cytokine production in rats [57]. Pregnant women with preeclampsia had similar levels of circulating 1,25(OH)_2_D compared to controls. In culture, placentas from preeclamptic women secreted higher concentrations of inflammatory cytokines, and lower anti-inflammatory IL-10 compared to placentas from women with a normal pregnancy. When the culture media was supplemented with vitamin D in hypoxic conditions (used to stimulate the production and secretion of pro-inflammatory cytokines), placental tissue from preeclamptic pregnancies secreted significantly lower concentrations of hypoxia-induced antiangiogenic factor, sFlt-1, and had diminished IL-6 production compared to preeclamptic placentas cultured without vitamin D supplementation [57].

To investigate the effect of vitamin D supplementation on blood pressure and circulating T cells, the rats induced by reduced uterine perfusion pressure (RUPP), a model of preeclampsia, were administered vitamin D_2_ or vitamin D_3_ on gestation days 14–18 [57]. Mean arterial blood pressure (MAP) and CD4+ (Please confirm if + is superscript) T cells were reduced in RUPP rats receiving vitamin D_3_ compared to non-supplemented RUPP rats. Vitamin D_2_ supplementation decreased circulating CD4+ T cells but had no effect on MAP. Similar improvements to systolic and diastolic blood pressure were observed in healthy pregnant women after a nine-week supplementation of 400 IU/day vitamin D_3_ reduction in comparison to placebo suggesting its potential use in the prevention of preeclampsia [45].

A randomized controlled trial in two South Carolina health centers provided either a 2000 IU/day or 4000 IU/day vitamin D_3_ supplement in the 12th–16th week of gestation until delivery [58]. A mean change in baseline 25(OH)D was greater in the 4000 IU/day group with 46.2% of participants reaching 40 ng/mL prior to delivery, a level determined to achieve maximal 1,25(OH)_2_D conversion [59], compared to 37.4% in the 2000 IU/day group. An inverse association between late trimester mean 25(OH)D levels with maternal infections and preterm delivery was reported with no cases of hypercalciuria or hypercalcemia. The authors concluded that these links are only suggestive since there was no difference in pregnancy complication risk among the vitamin D intervention groups. It is important to note that this study did not have a placebo or control group to minimize or adjust the effects of other variables on pregnancy complications.

When the data were combined with a concurrently run vitamin D intervention trial (NICHD; National Institute of Child Health and Human Development) by the same group, there was a marginally significant reduction in hypertension disorders (*p* = 0.052) after adjustment for race and study in the 4000 IU/day group when compared to control women receiving 400 IU/day [60]. Using maternal 25(OH)D concentrations as the outcome, for every 10 ng/mL increase in 25(OH)D at delivery, there was a trend of reduced odds ratio for infections, preterm birth without preeclampsia, and hypertension disorders of 0.89, 0.84, and 0.77, respectively after adjustments for study and race. Combining the main comorbidities, for every 10 ng/mL increase in 25(OH)D at delivery, the odds ratio was reduced to 0.84 (*p* = 0.006) indicating fewer adverse pregnancy outcomes as maternal vitamin D levels increased.

Hossian *et al.*, provided 4000 IU vitamin D_3_/day or a placebo to women starting at 20 weeks of gestation until delivery [61]. While serum vitamin D levels improved in the intervention group, only 15% of the women obtained levels of ≥30 ng/mL (Endocrine Society cut-off for vitamin D sufficiency) due to severely low baseline 25(OH)D levels in this population (3–9 ng/mL). Thus, it is not surprising that this study found no effect of the vitamin D supplementation on incidences of preterm birth, preeclampsia, or small for gestation age since 23% of participants had persistent serum 25(OH)D below 10 ng/mL which is considered vitamin D deficiency status by both the IOM and Endocrine Society (Table 1). Sablok *et al.*, provided vitamin D supplement doses depending on baseline level of serum 25(OH)D levels or placebo [62]. Women with deficient levels (classified by the authors as <25 nmol/L) received 120,000 IU D_3_ at 20, 24, 28, and 32 weeks gestation, whereas women with insufficient levels (25–50 nmol/L) received 120,000 IU at 20 and 24 weeks and sufficient women (>50 nmol/L) received one dose of 60,000 IU at 20 weeks. Infants of mothers receiving vitamin D supplementation were more likely to reach vitamin D sufficiency at birth compare to infants of non-supplemented mothers (46.2% *vs.* 14%, respectively), however, over half of the infants in the supplement group still had suboptimal vitamin D status. Rates of preterm birth and hypertension were lower in the supplemented groups compared to non-supplemented (21.1% *vs.* 8.3%, 21.1% *vs.* 11.1%, respectively), however, rates of hypertension did not reach significance (*p* = 0.08). Preeclampsia is a common pregnancy complication and a major contributor to maternal mortality worldwide. Vitamin D supplementation is a low cost intervention strategy and additional clinical trials powered to determine the effects of vitamin D on preeclampsia prevention and treatment are clearly warranted.

## 5. Infant

Epigenetic effects have been postulated as one of the mechanisms involved in developmental programming. Epigenetic changes reflect post-translational modifications to DNA including methylation, phosphorylation, and acetylation of histones without a change in DNA sequence, which ultimately regulate gene expression. Through VDR, vitamin D is involved in modulation of histone acetylation and methylation resulting in the activation or silencing of genes [63,64,65]. It has been proposed that vitamin D-induced epigenetic changes during development are involved in susceptibility to multiple morbidities ranging from allergies to cancer later in life. Thus it could be argued that adequate maternal-fetal transfer of vitamin D in utero and early infancy is important for life-long optimal health.

Breast milk is low in vitamin D concentration and is the primary source of vitamin D in breastfed infants [36] as it is recommended that infants younger than 6 months be kept out of direct sunlight and the exposure to sun be minimized with the use of protective clothing, hats and sunscreen [66]. Not only can seasonal variation impact cutaneous production in the infant, but also seasonal variation in maternal UVB exposure will impact breast milk vitamin D concentrations. If women plan to exclusively breast feed their baby, it is important for mothers to have adequate vitamin D status. Infant formula is supplemented with 400 IU/L, nevertheless, infants might not consume a liter of formula per day hence additional supplementation is recommended for breastfed infants and infants consuming less than one liter of formula per day by the American Academy of Pediatrics, the IOM [67], and the Endocrine Society [3] to ensure a total dietary intake of at least 400 IU per day.

### Respiratory Health and Allergic Disease in Children

Over the last decade, there is conflicting data on the role of maternal vitamin D on offspring allergic outcomes. Higher maternal vitamin D intake during pregnancy was found to be inversely associated with asthma [68], allergic rhinitis [68], wheezing [69], and eczema in early childhood. Using dietary intake data alone can be problematic since UVB-induced skin formation of vitamin D can significantly alter serum 25(OH)D, thus, dietary intakes might not be a reliable indicator of overall vitamin D status. Jones *et al.*, reported that low cord blood 25(OH)D levels were associated with development of eczema in infants at six and twelve months, but not at 30 months (*p* = 0.12) [70]. While there was no relationship between cord blood 25(OH)D and wheezing at any time point in this study, it is suggestive that early life development could have an impact on allergic phenotype later in life. 25(OH)D levels were independently associated with an increased risk of food allergen sensitization and severity of dermatitis in infants with atopic dermatitis [71]. The number and degree of food allergen sensitizations inversely correlated with 25(OH)D levels as measured by IgE-specific levels. Complicating these relationships are the findings that high maternal 25(OH)D levels are associated with an increased risk of childhood allergic diseases [72,73,74]. The Tucson Infant Immune Study observed a U-shaped curve of cord blood 25(OH)D less than 50 nmol/L and levels higher than or equal to 100 nmol/L had higher total and inhalant allergen-specific IgE levels compared to children with 25(OH)D levels between 50 and 99.9 nmol/L [74].

One randomized controlled trial investigated the effects of prenatal vitamin D supplementation on respiratory outcomes in children at three years of age. Women were asked to avoid use of vitamin D supplements and were randomized at 27 weeks gestation to receive 800 IU/day vitamin D_2_ until delivery, a single oral bolus of 200,000 IU vitamin D_3_, or no intervention [75]. No differences in the prevalence of eczema, atopy, or wheezing were found among the groups in children at 3 years of age. Similar results were found when allergy indicators were analyzed based on cord blood 25(OH)D levels. Additional data collected on lung function, total serum IgE levels and eosinophil counts also showed no significant differences, contrasting with the study by Baek *et al.* [71]. However, only a small percentage of the children participated in this additional follow-up, which may have contributed to a lack of statistical power to be able to detect differences among the groups.

Although cord blood 25(OH)D levels were significantly higher in the supplemented children compared to the control group, only a small percentage of offspring receiving daily vitamin D (13%) or bolus vitamin D (3%) had cord blood vitamin D levels ≥ 50 nmol/L. An ongoing supplementation trial (ABCvitaminD) is providing a higher dose vitamin D_3_ supplement of 2400 IU/day or placebo from 24 weeks of gestation until 1 week post-delivery. Children will be followed for three years for respiratory health outcomes and the data will provide a better look into the effects of a higher dose vitamin D supplement on allergic outcomes.

Nutrient interventions beginning in the third trimester might miss the critical window of immune developmental programming. In an on-going trial (Vitamin D Antenatal Asthma Reduction Trial, VDAART) the influence of vitamin D supplementation beginning in the second trimester on different infant allergic disease risk at three years of age is being explored [76]. Women are being supplied daily with 4000 IU of vitamin D_3_ plus a multivitamin containing 400 IU vitamin D_3_, or a daily placebo plus the 400 IU vitamin D_3_ until delivery. Ancillary outcomes being followed include maternal vaginal flora and maternal and infant intestinal flora. This study plans to follow children up to six years of age, which is the age when most asthma diagnoses are classified. It is anticipated that this study will provide valuable new information on the effects of maternal vitamin D status and offspring allergy/asthma outcomes.

Grant *et al.*, reported that supplementing both mother/infant pairs with vitamin D from the third trimester to 6 months postnatal reduced the amount of primary care visits for acute respiratory infection [77]. Infants in the higher dose supplement group (mothers received 2000 IU: infants received 800 IU per day) had a lower proportion of child primary care visits compared to placebo (87% *vs.* 99%, respectively, *p* = 0.004). The lower dose supplement group (mothers received 1000 IU: infants received 400 IU per day) had similar proportion of acute respiratory infection visits as the placebo group (95%, *p* = 0.17). Since the study looked at the number of visits rather than confirmed diagnosis of infection, this study suggests infant vitamin D supplementation in addition to prenatal supplementation might be beneficial in reducing health care visits for acute respiratory infections.

The data concerning vitamin D treatment on the clinical severity of atopic dermatitis has been more consistent. Between the months of February and March, supplementation of 1000 IU/day of vitamin D_3_ or placebo was randomized to children age 2–17 with winter-related atopic dermatitis [78]. After the one month, there was a significant improvement in clinical severity of winter-related atopic dermatitis in the vitamin D group compared to placebo. These results are in agreement with other intervention trials [79,80]. Samochocki *et al.*, supplemented 2000 IU/day vitamin D_3_ for 3 months in adult subjects with atopic dermatitis and reported a significant reduction in the severity of atopic dermatitis and total serum IgE levels compared to baseline [79]. Prior to supplementation, the frequency of bacterial infections was higher in atopic dermatitis patients with 25(OH)D levels <30 ng/mL compared to those with levels ≥30 ng/mL [79]. Despite the short supplementation duration of three months; there was no report of bacterial infection during the treatment period. While the results look promising, placebo controls were not included in this study. In contrast to the above trial, Hata *et al.*, found no change in Eczema Area and Severity Score (EASI) or IL-13 expression after a 21-day supplementation of 4000 IU/day vitamin D_3_ [81].

## 6. Risk of Toxicity

The causes of vitamin D intoxication from high dietary intakes can result from errors in fortification dosages, manufacturing mistakes of over-the-counter supplements, or misunderstanding doctor recommendations. The long half-life and storage of 25(OH)D in fat may pose problems with regard to vitamin D toxicity. During intoxication, high levels of 25(OH)D and 1,25(OH)_2_D can lead to increased absorption of calcium in the intestine causing hypercalcemia. Increased pressure on the kidneys to filter the excess calcium leads to hypercalciuria, and if prolonged, polyuria and dehydration can occur due to an inability to regulate urine concentrations [82]. The IOM and Endocrine Society differ on the cut-off levels that represent the Upper Limit or a risk of toxicity e.g., >50 ng/mL and >150 ng/mL, respectively. It is important to note, that these concentrations represent a risk for toxicity rather than levels at which toxicity signs will definitively occur. The incidence of 25(OH)D levels above 50 ng/mL (toxicity cut-off for IOM) in the Rochester Epidemiology Project from 2002 to 2011 increased from 9 to 23 cases per 100,000 [83]. Out of 1070 patients with serum vitamin D levels > 50 ng/mL, 15% (165) of patients presented with hypercalcemia. However, when regression analysis was performed, 25(OH)D levels and serum calcium levels had no significant relationship (*p* = 0.20). Still, some professionals fear that with the increase in supplementation rates, there could be a rise in vitamin D toxicity. Since the Food and Drug Administration (FDA) does not heavily regulate supplements, supplement manufacturing can be a cause of concern. Five out of fifteen over-the-counter vitamin D supplements in the United States were found to have amounts of vitamin D outside a 90%–120% variability range of the stated quantity on the bottle [84]. As mentioned earlier, genetics can play a role in determining an individual’s vitamin D status. Common gene polymorphisms in 7-dehydrocholesterol reductase, CYP2RI, and vitamin D binding protein were found to significantly alter serum 25(OH)D levels [85]. Loss of function of 24-hydroxlase (CYP24A1), the enzyme responsible for the breakdown of 1,25(OH)_2_D and 25(OH)D, can increase the risk of vitamin D toxicity due to a reduced ability to inactivate these vitamin D compounds in the body [86].

### 6.1. Potential Consequences of Excess Vitamin D Intakes: Maternal and Infant

During pregnancy, the current recommended dietary intake of vitamin D and upper limit ranges from 200 to 2000 IU/day and 3200 to 10,000 IU/day, respectively (Table 2). Dawodu *et al.*, supplemented a cohort of vitamin D deficient women with varying doses of vitamin D_3_: 4000 IU/day, 2000 IU/day, or 400 IU/day starting at 12–16 weeks of gestation to delivery [87]. Supplementation with 4000 IU/day was found to be the most effective in achieving serum levels ≥ 32 ng/mL at delivery (65%, 24%, and 10%, for the 3 supplement groups, respectively) and cord blood levels ≥ 20 ng/mL (79%, 44%, and 21%, respectively). No adverse outcomes were observed in any groups as measured by serum and urine calcium levels. These results are in accordance with previous randomized controlled trials in mixed demographic of ethnicities [58,59,88]. Roth *et al.*, reported serum 25(OH)D ≥ 130 ng/mL at delivery in Bangladesh women following 35,000 IU/week supplementation of vitamin D_3_ in the third trimester [89,90]. Women in this study had average baseline 25(OH)D levels above sufficiency cut-offs (IOM and Endocrine Society guidelines) and had no signs of adverse effect reported whilst observing a positive effect on infant growth outcomes.

Since pregnancy is a relatively small window of time, maternal adverse side effects might not occur within the potential ten months of supplementation. Data regarding long-term high dose vitamin D supplementation in women of childbearing age are lacking. Similarly, studies are needed to address whether women who are vitamin D sufficient at conception and receiving high doses of vitamin D (≥4000 IU/daily) have adverse pregnancy outcomes. Also, studies powered to determine the effect of separate ethnic groups’ varying baseline vitamin D levels on 25(OH)D levels after different vitamin D supplementation doses will provide more concrete information for individualized recommendations during pregnancy.

A recent six month randomized controlled trial of 1200 IU/day in breastfeeding women resulted in a significant increase in 25(OH)D levels compared to women receiving 400 IU/day, but only 25% of the women taking 1200 IU/day reached levels above 30 ng/mL, the Endocrine Society cut-off for sufficiency (Table 1) [91]. The women in both groups had mean baseline levels considered vitamin D deficient thus supplementing higher amounts than 1200 IU/day would be needed to achieve sufficiency for this population. Oberhelman *et al.*, provided daily supplements of 5000 IU/day for 28 days or one time dose of 150,000 IU to healthy women who were exclusively breastfeeding [92]. Both doses successfully raised maternal and infant 25(OH)D levels [92]. The single dose was able to raise levels at a quicker rate but no difference in serum concentrations were found at 28 days in mother or infant. Four women in the daily dose group and three women in the single dose group had urine calcium/creatinine ratios above the accepted reference range, while no changes in serum calcium were observed. Although both single and daily doses of vitamin D_3_ seem to be safe, caution should be considered when supplementing at these high doses in pregnant women, particularly if baseline vitamin D status is sufficient.

A one-time, oral bolus of vitamin D is an effective way to treat vitamin D deficiency as 25(OH)D levels quickly rise following supplementation. However, the question of toxicity risk following a large, bolus should be considered. Recommendations for the upper limit for vitamin D intake ranges from 3200 to 10,000 IU (Table 2). It is not completely known how large, acute increases in circulating 25(OH)D levels immediately following supplementation can affect a fetus/infant, who are vulnerable to vitamin D toxicity at this time in development. As previously mentioned, short-term studies generally agree with its safety but long-term studies are needed to address the question of whether large bolus vitamin D supplementation during pregnancy is associated with any adverse health outcomes in the offspring later in life.

### 6.2. Infant Toxicity

Gallo *et al.*, designed a study to determine an oral dosage of vitamin D that would achieve a 25(OH)D level of 75 nmol/L or greater in 97.5% of newborn, breastfed infants by three months of age [93]. Healthy newborns, less than one month of age and consuming at least 80% of total milk volume from breast milk, were supplemented with different dosages (400, 800, 1200, and 1600 IU/day vitamin D_3_) for 11 months [93]. The 1600 IU vitamin D_3_/day is four times the IOM recommendations for newborns. This high-dose group was discontinued early because 93% of infants had plasma 25(OH)D levels ≥ 250 nmol/L, which was reported to be associated with hypercalcemia (IOM, 2011 report). By 3 months, 97% of infants from any of the four supplement groups achieved 25(OH)D levels above ≥ 50 nmol/L with no differences between groups. Differences among the groups were observed when looking at the number of infants achieving serum 25(OH)D levels ≥ 75 nmol/L. By three months, a higher percentage of infants in the 1200 and 800 IU/day groups reached blood levels ≥ 75 nmol/L (92% and 81% respectively) compared to 400 IU/day (55%). Higher vitamin D supplement arms were able to sustain higher infant vitamin D serum levels at 12 months when compared to 400 IU/day, which agrees with a study by Grant *et al.* [94]. However, it is important to note that additional safety monitoring should be done when supplementing with high dose vitamin D_3_ in newborns, as hypercalcemia and hypercalciuria are potential adverse effects.

Although numerous cases of newborn vitamin D toxicity have been reported over the past 80 years, toxicity is rare when compared to the number of newborns receiving vitamin D supplementation [95,96,97,98]. A recent case report on an exclusively breastfed four-month old infant receiving over the counter liquid vitamin D supplementation showed 25(OH)D levels of 294 ng/mL, severe hypercalcemia, hypercalciuria, and nephrocalcinosis [95]. The toxicity symptoms exhibited in the infant included irritability, dehydration, anorexia, and gastrointestinal issues. The toxicity was the result of the mother providing a higher dose (dropper full per day *vs.* the recommended one drop per day) of the supplement to the infant for two months. In addition, the amount of vitamin D in the supplement was threefold higher than what was listed on the label. It was estimated that the infant was receiving 50,000 IU daily for a two-month duration. After 89 days off of the supplement, 25(OH)D levels lowered to 54 ng/mL and normal growth and developmental milestones were being reached, however, nephrocalcinosis was not reversible. Levels as high as 470 ng/mL have been reported in infant toxicity cases and have life-long consequences, especially adverse renal function, or even death [98]. It is important to note that the toxicity signs presented above were not seen in any recent supplementation trial and the causes of these intoxications occurred with infants/children receiving doses from 50,000 to 600,000 IU. Also, loss of function mutations in 24-hydroylase was found to be a risk factor for idiopathic infantile hypercalcemia making those infants more susceptible to vitamin D toxicity even at lower vitamin D supplementation doses [86], thus genetics can play a role in both vitamin D deficiency and toxicity.

## 7. Summary and Challenging Issues

Appropriate Vitamin D dietary recommendations are a subject of intense debate [99]. With large percentages of individuals having insufficient vitamin D levels, this raises the question of whether fortification efforts should be increased. Many countries currently mandate the fortification of certain foods with vitamin D, e.g., 100 IU/cup milk in the United States, 30–45 IU/ 100 mL milk in Canada, and edible oil spreads (margarine) in Australia, however, a large proportion of dietary intakes are still below recommended levels [100,101]. Using NHANES data, 78% of women of childbearing age did not meet the EAR for vitamin D (*n* = 3210) [102]. A recent report by the 2015 Dietary Guidelines Advisory Committee noted that even with careful food choices and following recommended amounts from each food group in the (current) USDA food patterns, the RDA for vitamin D cannot be met [103]. Their conclusion: “Through the use of a diet rich in seafood and fortified foods, EAR, but not RDA, levels of vitamin D can be achieved. Additional fortification or supplementation strategies would be needed to reach RDA levels of vitamin D intake consistently, especially in individuals with low intakes of fish/seafood or fortified dairy foods, other fortified foods (e.g., breakfast cereals) and beverages”.

Unlike folic acid fortification of grain products, vitamin D fortification has primarily focused on dairy products, potentially missing certain populations due to cultural practices or perceived or actual lactose intolerance. Many public health officials have proposed the need for enrichment of other foods. Mushrooms grown under UVB light can increase vitamin D_2_ levels and successfully raise 25(OH)D in vegetarians or non-dairy consuming individuals [104]. Hens fed diets high in vitamin D can increase the amount of vitamin D_3_ in the yolks from 50 IU to over 2000 IU per egg without causing harm to the hen or egg quality [105].

The Food and Drug Administration (FDA) of the United States has proposed changes to the nutrition facts label that is found on most food packages. Among the changes, is the requirement that manufacturers declare the amount of vitamin D on the label, as it is one of the new “nutrients of public health significance” based on NHANES dietary intake data [106]. Vitamin D supplementation/fortification has also been met with opposition [107] due to concerns that vitamin D is a hormone and should be treated as such including addressing efficacy, dose and side effects. However, vitamin D is not a hormone but rather a pro-hormone. Other concerns are the insufficient evidence to determine the effects of vitamin D supplementation/fortification on bone health and fracture incidence and that FDA recommendations to include vitamin D on food labels may encourage fortification of vitamin D in products that would not otherwise necessarily be recommended, and lastly, that there is some evidence for possible adverse consequences to vitamin D fortification [107].

While the essentiality of adequate vitamin D during pregnancy is not disputed, controversy exists over the recommended dietary intake levels, the mathematical approaches to calculating that level, and the endpoints used to determine requirements; the concentrations of circulating 25(OH)D that denote deficiency, insufficiency and adequacy status; and whether race, body weight or other factors should be taken into account when making dietary recommendations, Similarly, it is not clear what vitamin D supplementation regimens are most effective during pregnancy; whether mother, child or both should be supplemented; and whether there are long-term consequences of supplementation. What is clear is that additional randomized, clinical trials that address these issues are warranted.
